# Development of Clinical Prediction Rules for One-Year Postoperative Functional Outcome in Patients with Intertrochanteric Fractures: The Intertrochanteric Fracture Ambulatory Prediction (IT-AP) Tool

**DOI:** 10.3390/ijerph19010177

**Published:** 2021-12-24

**Authors:** Nath Adulkasem, Phichayut Phinyo, Jiraporn Khorana, Dumnoensun Pruksakorn, Theerachai Apivatthakakul

**Affiliations:** 1 Department of Orthopaedics Surgery, Faculty of Medicine Siriraj Hospital, Mahidol University, Bangkok 10700, Thailand; adulkasem.n@gmail.com; 2 Center for Clinical Epidemiology and Clinical Statistics, Faculty of Medicine, Chiang Mai University, Chiang Mai 50200, Thailand; jiraporn.kho@elearning.cmu.ac.th; 3 Department of Family Medicine, Faculty of Medicine, Chiang Mai University, Chiang Mai 50200, Thailand; 4 Musculoskeletal Science and Translational Research (MSTR) Cluster, Chiang Mai University, Chiang Mai 50200, Thailand; dumnoensun@hotmail.com; 5 Division of Pediatric Surgery, Department of Surgery, Faculty of Medicine, Chiang Mai University, Chiang Mai 50200, Thailand; 6 Department of Orthopaedics, Faculty of Medicine, Chiang Mai University, Chiang Mai 50200, Thailand; tapivath@gmail.com

**Keywords:** fracture fixation, geriatric, intertrochanteric fractures, clinical prediction rules, functional outcome

## Abstract

Individualized prediction of postoperative ambulatory status for patients with intertrochanteric fractures is clinically relevant, during both preoperative and intraoperative periods. This study intended to develop clinical prediction rules (CPR) to predict one-year postoperative functional outcomes in patients with intertrochanteric fractures. CPR development was based on a secondary analysis of a retrospective cohort of patients with intertrochanteric fractures aged ≥50 years who underwent a surgical fixation. Good ambulatory status was defined as a New Mobility Score ≥ 5. Two CPR for preoperative and intraoperative predictions were derived using clinical profiles and surgical-related parameters using logistic regression with the multivariable fractional polynomial procedure. In this study, 221 patients with intertrochanteric fractures were included. Of these, 160 (72.4%) had good functional status at one year. The preoperative model showed an acceptable AuROC of 0.77 (95% CI 0.70 to 0.85). After surgical-related parameters were incorporated into the preoperative model, the model discriminative ability was significantly improved to an AuROC of 0.83 (95% CI 0.77 to 0.88) (*p* = 0.021). The newly-derived CPR enable physicians to provide patients with intertrochanteric fractures with their individualized predictions of functional outcome one year after surgery, which could be used for risk communication, surgical optimization and tailoring postoperative care that fits patients’ expectations.

## 1. Introduction

Intertrochanteric fracture is considered one of the most concerning health conditions in the elderly population. Over the years, the incidence of this fracture type has increased concordantly with aging [1]. Patients with intertrochanteric fractures can be treated with either an operative or non-operative approach. However, since operative treatment was proven to be significantly superior in reducing morbidity and mortality compared to non-operative treatment, it is currently recommended as the standard treatment for intertrochanteric fracture [2]. Unfortunately, approximately half of the patients who underwent surgical operation with an uneventful fracture healing still had a poor ambulatory status [2].

As not all patients would achieve good ambulatory status after surgery, prediction of postoperative functional outcomes is clinically important and might be helpful for both physicians and patients in finding common ground on the postoperative care plan (e.g., deciding whether an intensive postoperative rehabilitation program should be prescribed, or an early discharge plan should be considered instead) [3,4]. Several preoperative factors were identified to predict treatment outcomes in patients with intertrochanteric fractures, such as patients’ baseline condition and pre-injury ambulatory function [5,6]. Some intraoperative parameters, such as reduction alignment and implant position, have previously been shown to be significant in predicting fixation failure [7,8].

Although these prognostic factors were associated with postoperative treatment outcomes, they were usually considered separately. In other words, they could not provide individual predictions, which would be more practical and relevant in clinical practice. One solution is to create clinical prediction rules (CPR) using statistical modeling, which would utilize the information from several predictors for estimating the probability, or the likelihood, of postoperative outcomes specifically for each patient [4]. For patients with intertrochanteric fractures, individualized predictions might be beneficial in at least two circumstances. First, primary care physicians or emergency physicians, who were usually the first to encounter the patients, could provide a rough estimation of postoperative outcomes to the patients and their families using only preoperative information [9]. Second, orthopaedic surgeons could use both preoperative and intraoperative information to predict individual treatment outcomes and use these predictions as guidance during fracture reduction and fixation adjustment to achieve satisfactory treatment outcomes [10].

Currently, only a limited number of CPR were available to predict functional outcomes in patients with intertrochanteric fractures [11,12]. In addition, most of the tools were developed to predict the ability to withstand mechanical failure after fracture fixation, or fixation failure [10,13,14]. Even though fixation failure is an important endpoint that should not be overlooked, predicting postoperative functional outcomes is also clinically relevant and might be more straightforward to the patients [15,16]. Thus, we intended to develop CPR using preoperative and intraoperative information to predict patients’ ability to ambulate one year after surgery, a clinically relevant functional outcome.

## 2. Materials and Methods

### 2.1. Study Design

Prognostic prediction research was conducted based on a secondary analysis of a retrospective cohort of patients with intertrochanteric fractures who received an operative treatment at a university-affiliated medical center from January 2017 to February 2020. The Institutional Review Boards approved the study protocol (No. 101/2021 study code ORT-2564-07985). All patients provided verbal informed consent to the investigators prior to the study inclusion.

### 2.2. Study Patients

All patients with intertrochanteric fractures aged ≥50 years who underwent surgical fixation at our institution during the pre-defined study period were included. We only included patients aged ≥50 years in the analysis, since half of the patients within this age group suffered from fragility hip fracture [17]. The exclusion criteria were patients with injuries other than low-energy trauma, patients with concomitant or previously injured extremities, which could interfere with the outcome interpretation, such as polytraumatized patients, patients with concomitant or previous major ipsilateral lower extremity injury, and patients diagnosed with pathological fractures. In addition, patients who could not provide clinical endpoint information or could not be reached by telephone call (e.g., passed away) were also excluded.

### 2.3. Candidate Predictors

For preoperative prediction, four candidate predictors were preselected and retrieved from electronic medical records, which were sex [18], body mass index (BMI) [19], Charlson comorbidity index (CCI) [6], and pre-injury ambulatory status [20]. The selection of predictors was based on the availability of information at the first patient encounter (e.g., primary care unit or emergency department). Preoperative laboratory parameters, including hemoglobin [21] and albumin level [22] at the time of surgery, were also retrieved.

For intraoperative prediction, several surgical-related parameters were preselected and documented: fracture configuration classified according to AO/OTA classification, lateral wall thickness measured from preoperative plain radiographs [23,24]. Fixation implants were categorized into intramedullary and extramedullary devices. Post-reduction alignment and implant position was determined using surgical-related parameters, including neck-shaft angle (NSA) [13], fracture displacement (medio-lateral and antero-posterior) [14], calcar-referenced tip-apex distance (CalTAD), and Parker’s ratio in AP view (the ratio representing the position of the proximal fixation in the coronal alignment) [8] from immediate postoperative plain radiographs. The depth of proximal fixation determined by CalTAD, as well as the cephalo-caudal position of the proximal fixation determined by Parker’s ratio, improve the strength of the fixation [8]. These surgical-related parameters were hypothesized to be the precursor of the postoperative ambulatory status [25].

The transparent reporting of a multivariable prediction model for an individual prognosis or diagnosis (TRIPOD) statement recommends against categorization of continuous variables to prevent information loss [26]. Hence, all laboratory profile and radiographic measurement variables were kept as continuous data. The actual shape of the association between the continuous predictors and the outcome of interest was illustrated using fractional polynomial plots and locally weighted scatterplot smoothing (LOWESS).

### 2.4. Study Endpoints

Good ambulatory status was defined as a New Mobility Score (NMS) ≥ 5 according to Parker et al. [27]. The NMS was generally used for ambulatory status assessment one year after surgery. The NMS consists of four levels of ambulation independence in three different situations. The score ranges from 0 to 9. In this study, a structured telephone interview was used for obtaining pre-injury NMS, NMS at the time of discharge, and one-year postoperative NMS. The inter-observer reliability of the score has been proven with an intraclass correlation of 0.98 [28]. The interview was performed by investigators who were blinded to the patients’ clinical information to minimize interviewer bias [29].

### 2.5. Statistical Methods

#### 2.5.1. Fundamental Statistical Analysis

All statistical analyses were computed using STATA (version 17.0, StataCorp LLC., College Station, TX, USA). Continuous variables were presented according to the distribution pattern. Continuous data were presented by means and standard deviation (SD) for normally distributed data. At the same time, median and interquartile range (IQR) was used for non-normally distributed data. Continuous data were tested using independent *t*-test and Man–Whitney U test as appropriate. Categorical data were described using frequency and percentage and compared using Fisher’s exact probability test. Statistical significance was defined as a *p*-value less than 0.05.

#### 2.5.2. Model Development

##### Study Size Estimation

The study size was estimated based on the standard recommendation of 10 events of interest per one included predictor [30]. Therefore, 120 good postoperative ambulatory status intertrochanteric fracture patients were expected for modeling of 12 predictors.

##### Multivariable Fractional Polynomial Modeling

Preselected candidate predictors were modeled using the multivariable fractional polynomial (MFP) method to avoid unnecessary categorization or inappropriate modeling of the determinants–outcomes association that violates the linearity assumption. The MFP function allows investigators to identify the most appropriate functional form for each included continuous predictor. Two steps in the MFP were described [31]. The first step of MFP is to eliminate all insignificant predictors from the model. In most cases, the alpha level would be set at 0.05 to exclude the insignificant terms. However, in our study, we preferred to preserve all preselected variables based on prior clinical knowledge and previous findings from the literature. In the second step, we determined the fittest fractional transformation for continuous covariates to be included during statistical modeling using a closed test algorithm [31].

**Figure 1:**
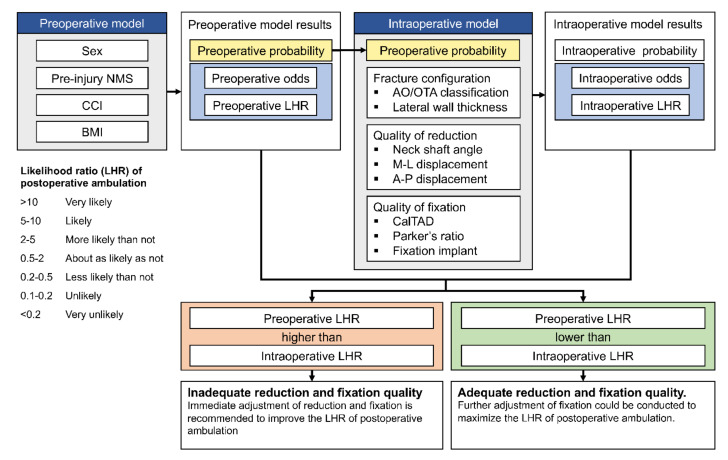
An infographic of the intertrochanteric fracture ambulatory prediction (IT–AP) tool. A preoperative model was modeled using the patient’s pre-injury demographic information, for which preoperative probability, odds, and likelihood ratio of postoperative functional outcome will be provided. After incorporating surgical-related parameters and the preoperative probability into an intraoperative model, an intraoperative prediction will be calculated and reported. A recommendation regarding the result provided by both preoperative and intraoperative prediction models will be given. NMS, New Mobility Score; CCI, Charlson comorbidity index; BMI, body mass index; LHR, likelihood ratio; M–L, medio-lateral; A–P, antero-posterior; CalTAD, calcar-referenced tip-apex distance.

In this study, we planned to develop two clinical prediction models to be used in two different clinical circumstances. The first model was the preoperative model, which was intended to be used during the preoperative period by attending emergency physicians or general practitioners to provide the patients and their families with preoperative predictions of the probability to ambulate after surgery. As the point of prediction took place before surgery, only the patient’s baseline functional status and preoperative clinical parameters were included in this model. The second model was the intraoperative model, which was intended to be used by the orthopaedic surgeon during the operation, specifically during reduction and fixation. Finally, for intraoperative prediction, surgical-related parameters were included together with the predicted probability of ambulation from the preoperative model (Figure 1).

##### Model Performance and Internal Validation

Model discriminative ability was measured using the area under the receiver operating characteristic curve (AuROC). When incorporated into the preoperative model, the added value of the surgical-related parameters was tested using the methods described by Delong and Clarke-Pearson [32]. Although the study base was technically cohort, excluding patients who could not be reached for NMS evaluation may give rise to selection bias, which might alter the precision of the predicted risk. Accordingly, this study presents the predicted odds from linear predictors instead of the predicted probability. The model calibration was presented with a calibration plot in addition to Pearson’s goodness-of-fit test. The calibration plot illustrates the agreement of the actual observed event and deciles of the predicted odds. The models’ optimism was evaluated using a bootstrap internal validation procedure with 200 replications. We also estimated the shrinkage factor to be used during external validation.

##### Model Interface and Clinical Implementation

The derived models were presented as an easy-to-use web application for clinical applicability: the intertrochanteric fracture ambulation prediction (IT-AP). Based on individual inputs, the application would estimate the predicted odds and the likelihood ratio (LHR) of having good ambulatory status one year after surgery (Figure 1). The LHR in this study is defined as the odds of having a good ambulatory status for each patient compared to the odds of having a good ambulatory status for an average patient with intertrochanteric fractures. Thus, when the LHR is higher than one, it means that the odds of having a good ambulatory status for that individual patient are higher than an average patient with intertrochanteric fracture. In contrast, when the LHR is lower than one, it means that the odds of having a good ambulatory status for that individual are lower than the odds of an average patient with intertrochanteric fracture. The IT-AP models, which utilize the information from both preoperative and intraoperative parameters, can assist orthopaedic surgeons during the surgery by comparing the values of preoperative LHR and intraoperative LHR. However, when the intraoperative LHR is less than the preoperative LHR, the surgeon should be cautious, as this indicates that the current reduction and fixation are likely to be suboptimal and immediate reduction and fixation adjustment are highly recommended.

## 3. Results

During the study period, 266 patients diagnosed with intertrochanteric fracture were included. Of those, we excluded 41 patients who have passed away, three patients who suffered from high energy injury mechanism, and one patient with pathological fracture. Finally, 221 patients were included, with 160 (72.4%) patients with an NMS ≥ 5. The comparison of baseline clinical characteristics, fracture configuration, and surgical-related parameters between patients with good and poor ambulatory status at one year is presented in Table 1.

**Figure 2:**
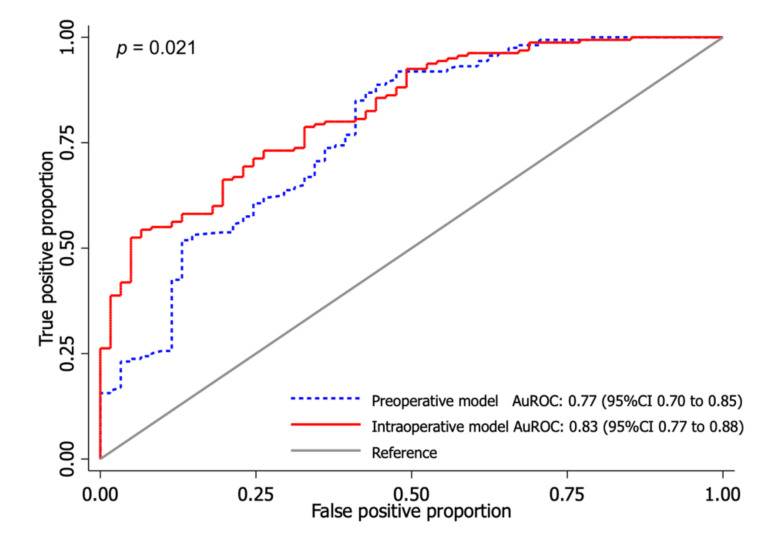
Comparison between area under the receiver operating characteristic (AuROC) curve of the preoperative (blue dash line) and intraoperative model (red line).

**Figure A1:**
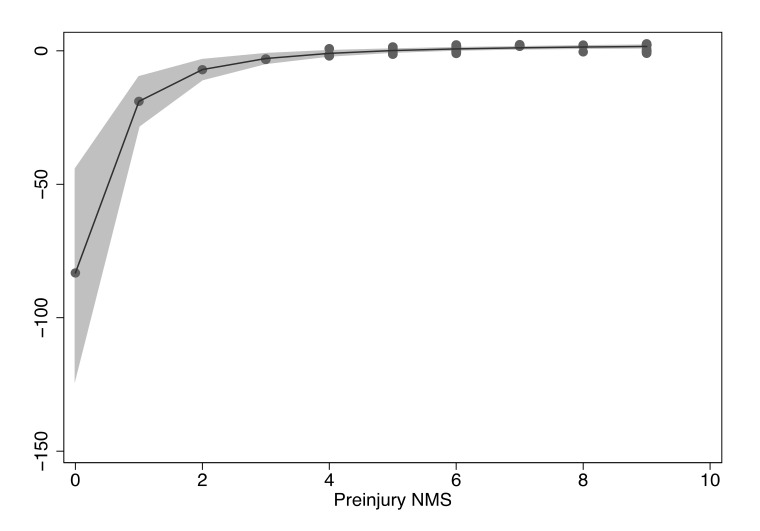
The shape of the association between ambulatory status and pre-injury NMS in the most fitted second-degree fractional polynomials term.

For the preoperative model, sex, BMI, CCI, and pre-injury NMS were included in the multivariable logistic regression. After applying the MFP algorithm, only the pre-injury NMS parameter was fitted and transformed into the second-degree fractional polynomial (FP2) terms. In contrast, the rest were fitted with the linear term (Appendix A: Figure A1). Table 2 presents the regression coefficient, 95% confidence intervals, and *p*-value of the transformed covariates. An AuROC of the derived model revealed an acceptable discriminative ability of 0.77 (95% CI 0.70 to 0.85) (Figure 2).

The intraoperative model was derived using the predicted probability from the preoperative model in combination with the preselected surgical-related parameters, including the AO/OTA classification, the lateral wall thickness, the NSA, the displacement, the CalTAD, Parker’s ratio, and the type of fixation implant. All predictors were fitted with linear terms without covariate transformation. The coefficients of each parameter with 95% CI and *p*-value are shown in Table 3. After incorporating surgical-related parameters into the preoperative model, the discriminative ability significantly increased by approximately 6% to an AuROC of 0.83 (95% CI 0.77 to 0.88) (*p* = 0.021) (Figure 2). This improvement represents the added discriminative value of surgical-related parameters to preoperative predictors.

**Figure 3:**
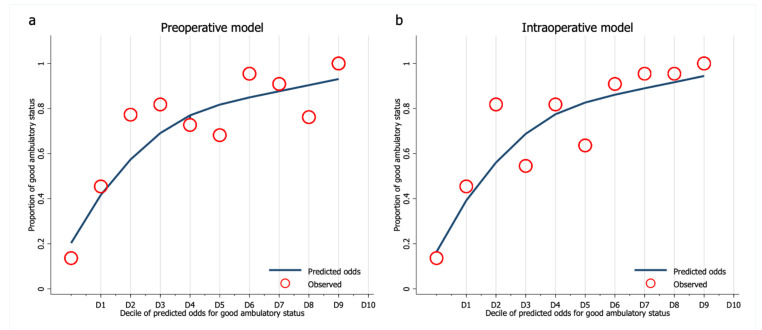
Calibration plot between an observed odds and decile divided predicted odds of (**a**) the preoperative model and (**b**) the intraoperative model.

The calibration of both models was presented with calibration plots (Figure 3). Pearson’s goodness-of-fit statistics were insignificant for preoperative and intraoperative models (*p* = 0.809 and *p* = 0.693, respectively). Internal validation with bootstrap re-sampling revealed an optimism of 0.01 (range −0.07 to 0.11) and 0.04 (range −0.04 to 0.14) with an estimated shrinkage factor of 0.92 (95% CI 0.90 to 0.94) and 0.80 (95% CI 0.78 to 0.82) for the preoperative and intraoperative model, respectively.

**Figure A2:**
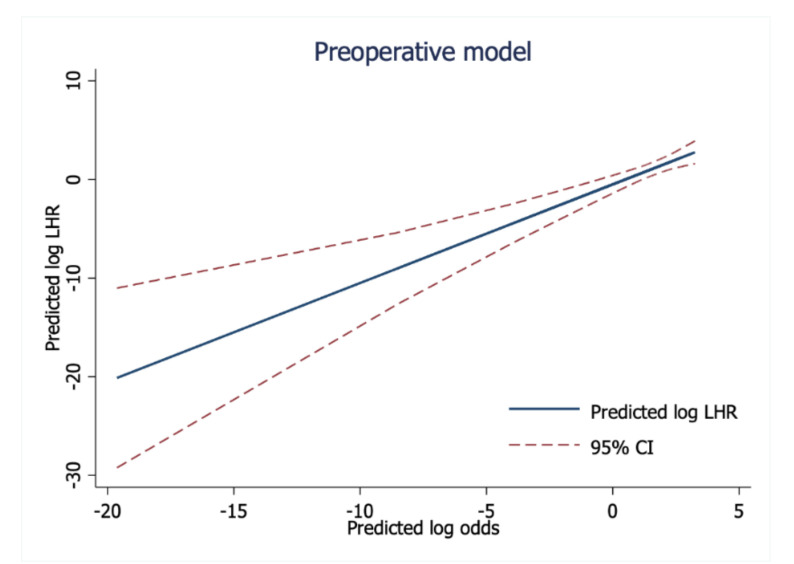
Predicted log-likelihood ratio (LHR) with 95% confidence interval of the preoperative model.

**Figure A3:**
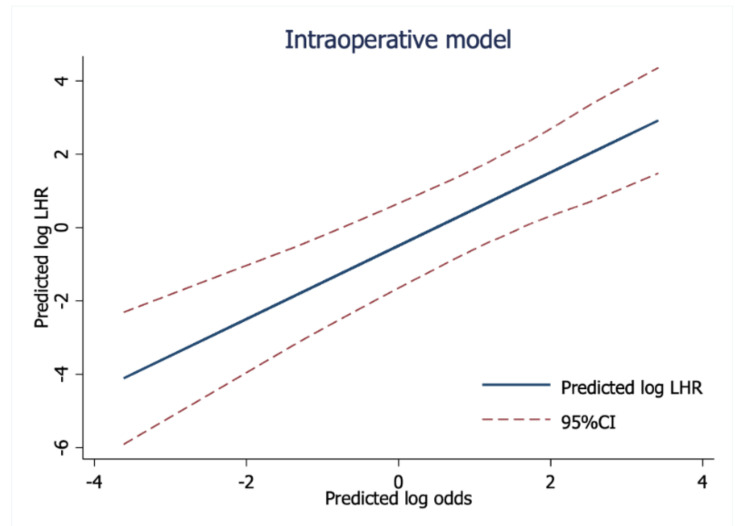
Predicted log-likelihood ratio (LHR) with 95% confidence interval of the intraoperative model.

The intertrochanteric fracture ambulation prediction (IT-AP) tool is available for use at: https://www.calconic.com/calculator-widgets/it-ap-tool/60596c43a9dbf0001ef1f4af (accessed on 15 November 2021). In addition, the predicted log LHR curves with 95% confidence intervals for both preoperative and intraoperative models are provided in Appendix B (Figure A2 and Figure A3). We also provided the values of the predicted LHR with 95% confidence intervals for each decile of predicted odds from the IT-AP model in Appendix B (Table A1 and Table A2).

## 4. Discussion

In the present study, we developed the CPR for both preoperative and intraoperative prediction of postoperative functional outcome at one year for patients with intertrochanteric fractures. The preoperative model consists of four predictors: sex, BMI, CCI, and pre-injury NMS. For the intraoperative model, surgical-related parameters, including fracture configuration, fracture reduction parameters, fixation quality, and type of implant, were added to the preoperative model. It was found that these intraoperative factors significantly improved the discriminative ability of the preoperative model in predicting postoperative ambulatory status at one year.

Previously, few CPR were developed to predict postoperative outcomes in patients with intertrochanteric fractures. Tanaka et al. developed a CPR for predicting declination of activity of daily living (ADL) at six months after operation [11]. The CPR was based entirely on patients’ clinical characteristics and did not consider the relevant surgical parameters. Subsequently, another CPR was developed by Murena et al. to predict implant failure using surgical-related parameters [10]. In this study, the IT-AP tool was developed to predict postoperative functional outcomes in terms of ambulatory status using both clinical and surgical-related parameters.

The IT-AP tool is composed of two models: the preoperative and intraoperative models. The preoperative model was proven to deliver an acceptably accurate prediction of the patient’s ambulatory status after surgery, which could be used by attending physicians for risk communication in primary or emergency care settings [33]. Within the preoperative model, the patient’s pre-injury ambulatory status was the strongest predictor of having a good postoperative functional outcome. Pre-injury ambulation ability was an independent predictor of functional outcome [7] and was also included in a previous CPR. In addition, as all predictors included within the preoperative model were chosen based on clinical availability, the model was highly pragmatic.

The intraoperative model was developed by incorporating surgical-related parameters with the predicted probabilities from the preoperative model. The model provides the likelihood of achieving a good one-year postoperative functional outcome, which can be used intraoperatively by orthopaedic surgeons in determining the current quality of reduction and fixation. All of the included parameters were preselected according to the previous literature [8,13,14]. An anatomical to a slightly valgus reduction of the fracture was associated with a high rate of fracture union [13]. The positive cortical reduction improves the fixation stability by limiting the fracture displacement [13,34].

Although each of the surgical-related parameters did not show statistically significant association with postoperative functional outcome, we included these parameters within our intraoperative model as it was previously proven to be effective in predicting fracture stability, which is one of the precursors of good postoperative functional status [25]. As a result, when surgical-related parameters were included with the preoperative predictions, there was a significant improvement in the discriminative ability of the intraoperative model. Our findings support the importance of considering both the patients’ initial clinical information and the intraoperative surgical parameters in predicting postoperative functional outcomes.

**Figure 4:**
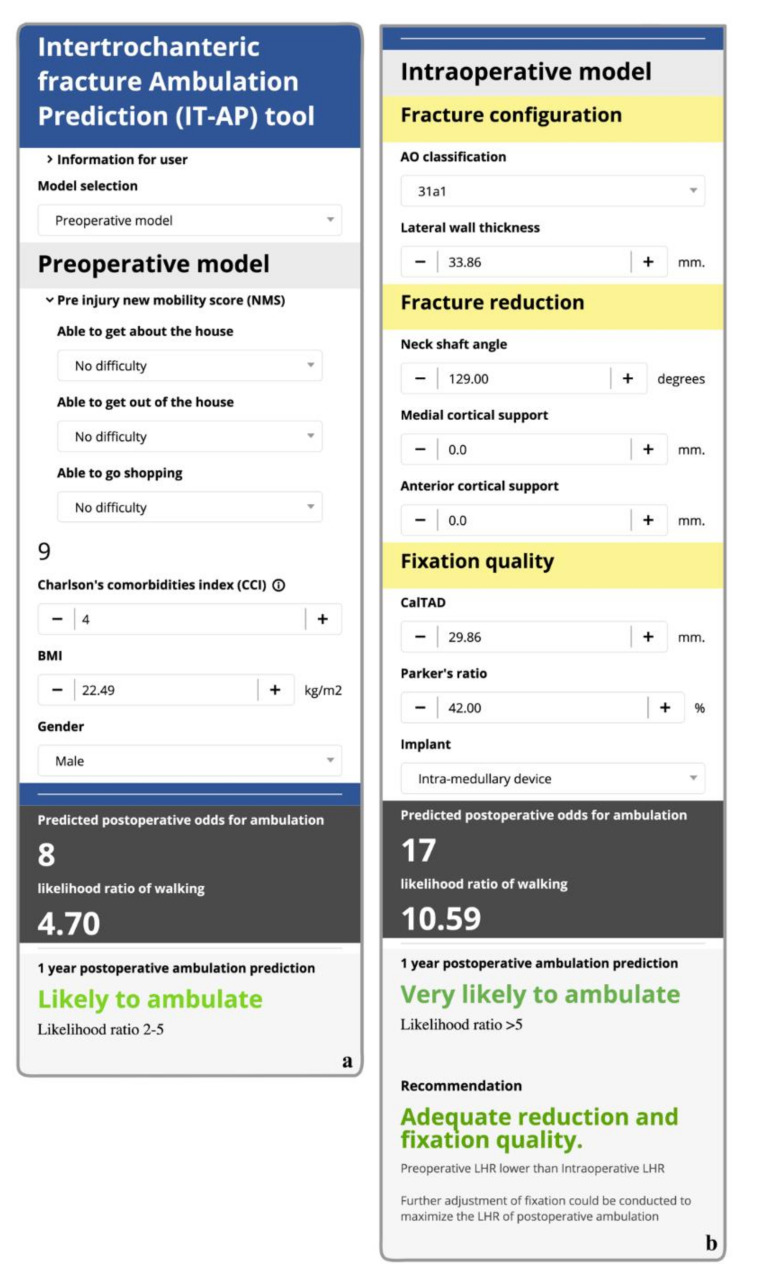
An interface of the Intertrochanteric fracture ambulation prediction (IT-AP) tool with an example information (**a**) preoperative model (**b**) intraoperative model.

**Figure A4:**
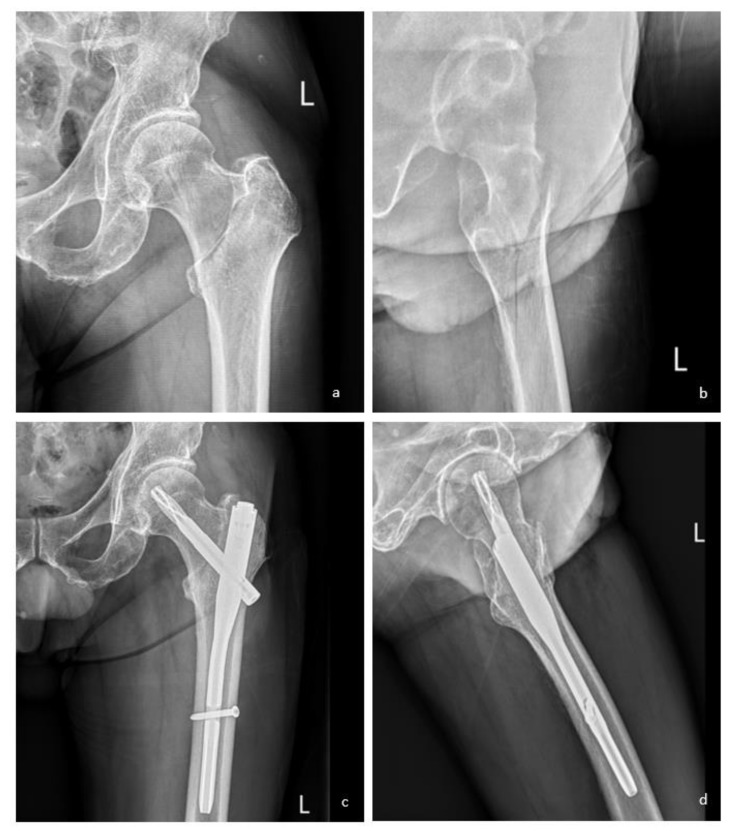
To demonstrate the use of the IT-AP tool, an example of 75-year-old male patient presented with left hip pain after a simple fall diagnosed as intertrochanteric fracture information was applied to the application. After the required data was inputted (pre-injury NMS = 9, CCI = 4, BMI = 22.49 kg/m^2^) in the IT-AP tool application under the preoperative model option, the application calculated the likelihood ratio of good ambulatory status after receiving operation (LHR+ = 4.7) and have been informed to the patient. The patient was then transferred to the secondary hospital under specialists’ care and scheduled for closed reduction and internal fixation with an intramedullary device. The fracture was classified as AO/OTA 31A1 with a lateral wall thickness of 33.86 mm (**a**,**b**). Right after fracture reduction with near anatomical reduction was conducted (NSA = 129, neutral reduction in both dimensions) and internally fixed (CalTAD = 29.86, Parker’s ratio = 42%) (**c**,**d**), the IT-AP tool revealed a likelihood ratio of 10.59 to which surgeon decided to proceed with the skin closure and finish the operation.

**Figure A5:**
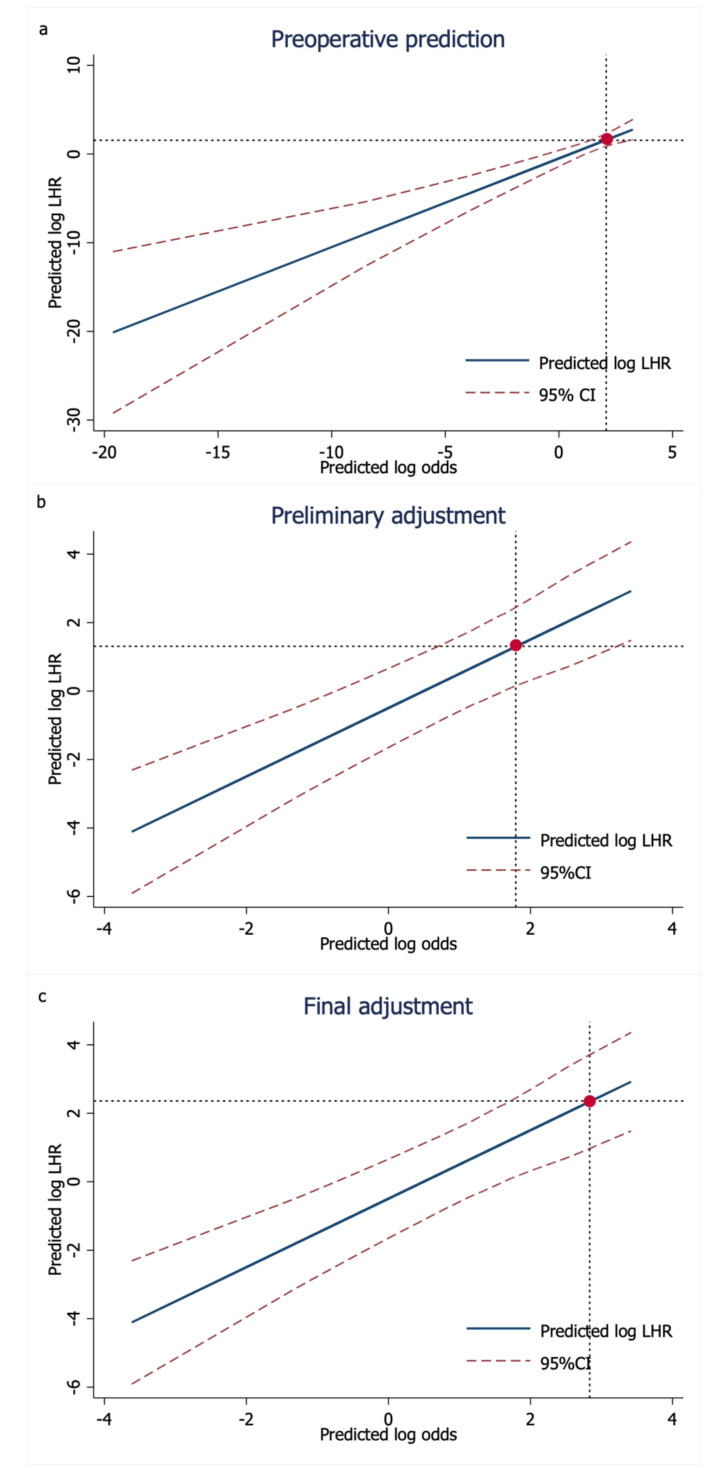
Predicted log-likelihood ratio (LHR) with 95% confidence interval of an example patient. (**a**) After applying preoperative information of an example patient from Table A3, the red circle marker demonstrates the predicted log-LHR with 95% CI. (**b**) The preliminary reduction and fixation demonstrated the declination of the predicted LHR and 95% CI. The results indicate that the quality of reduction and fixation was suboptimal and should be revised. (**c**) Improved predicted LHR and 95% CI were reported after an optimal reduction and fixation of the intertrochanteric fracture was achieved.

To demonstrate how the IT-AP tool would fit in clinical practice, we present a case of a 75-year-old male who presented with left hip pain and was subsequently diagnosed with an intertrochanteric fracture. To predict the likelihood of having good ambulatory status one year after the operation, the attending physician filled in all required predictor information into the IT-AP tool as shown in Figure 4 (Appendix C: Figure A4, Table A3). Then, the IT-AP tool under the preoperative model option estimated the LHR of having good ambulatory status after receiving operation at 4.7 (Appendix C: Figure A5a), which means that it was more likely than not that the patient would be able to ambulate at one year. In this case, the patient was scheduled for closed reduction and internal fixation with an intramedullary device (Appendix C: Figure A4a,b). After an initial adjustment, the orthopaedic surgeon wanted to know if the “current” adjustment was adequate. Surgical-related parameters were entered into the IT-AP tool under the intraoperative model option to estimate the LHR of ambulation considering fixation characteristics. The LHR was estimated at 3.70, representing a suboptimal surgical treatment (Appendix C: Figure A5b). The surgeon was then informed by the IT-AP tool that the “current” fixation and reduction characteristics were still inadequate and that further adjustment was still required (Appendix C: Figure A4c,d). Finally, after the surgeon performed additional surgical adjustments, the IT-AP tool revealed a LHR of 10.59, which was substantially higher than the predicted LHR from the preoperative model. Therefore, the surgeon decided to proceed with the skin closure and finish the operation (Appendix C: Figure A5c).

There are several strengths to this study. Our study was one of few studies to develop CPR for predicting the postoperative functional outcome [11]. The MFP algorithm preserves the nature of continuous predictors, which prevents significant loss of information [31]. Moreover, the simplified presentation of our CPR allows clinicians and specialists to calculate the individualized prediction for each patient easily. However, there are some limitations to be addressed. First, our CPR was developed using a relatively small cohort of patients, which may cause model overfitting. Nevertheless, only a minimal amount of model optimism was observed. Second, this study was susceptible to selection bias for excluding patients who passed away. For that reason, the study cohort might not be able to reflect the entire intended population, and therefore predicted odds, and a positive likelihood ratio, was reported instead of the predicted probability to avoid the misinterpretation of the predicted probability. However, it is important to note that the predicted odds, or the predicted LHR, from our model could overestimate the likelihood of ambulation. The degree of bias is unknown and needs to be proven in the validation study. Third, telephone interviews to collect outcome data may give rise to recall and interviewer biases. However, these biases were minimized by using a structured interview with a blinded interviewer [29]. Lastly, the apparent model performance reported might not generalize to other settings. Thus, external validation should be conducted before being implemented in practice.

While it is true that the surgery would be performed to the best of the surgeon’s ability, the definition of “the best” is not well described. In most cases, an acceptable range of an adequate quality of fixation and reduction varied among different surgeons [35,36]. From our perspective, the quality of surgery should be determined differently across each individual. An individualized prediction could help determine the most appropriate surgical-related parameters for each patient in order to achieve good postoperative ambulatory status. Therefore, we believe that our tool would benefit clinical practice by tailoring treatment for each patient.

## 5. Conclusions

The intertrochanteric fracture ambulation prediction tool, or the IT-AP tool, was developed to predict one-year postoperative functional outcomes in patients with intertrochanteric fractures. The tool consists of two statistical models developed for two specific circumstances: the preoperative and the intraoperative models. The preoperative model uses patients’ clinical information and baseline ambulatory status to provide preoperative predictions during the first encounter. In contrast, the intraoperative model uses surgical-related parameters to provide intraoperative prediction during surgical reduction and fixation. Orthopaedic surgeons could use the models’ prediction to assess the quality of current reduction and fixation parameters and whether an additional adjustment is required, potentially improving the overall quality of intertrochanteric fracture treatment and the patients’ functional outcomes.

## Figures and Tables

**Table 1:** Demographic data in 221 patients comparing between good functional outcome (NMS ≥ 5) and poor functional outcome (NMS < 5) and distribution after categorization.

Variable	NMS ≥ 5	NMS < 5	*p*-Value
	(160, 72.4%)	(61, 27.6%)	
Age (mean ± SD) years	80 ± 9	84 ± 7	0.002
Male (*n*, %)	49 (30.6%)	14 (23.0%)	0.318
Pre-fracture NMS ^1^ (median, IQR)	9 (7, 9)	6 (4, 9)	<0.001
Hb ^2^ (mean ± SD) g/dL	10.8 ± 1.6	10.2 ± 1.8	0.022
CCI ^3^ (median, IQR)	4 (4, 5)	5 (4, 6)	<0.001
BMI ^4^ (mean ± SD) kg/m^2^	21.5 ± 3.7	22.5 ± 3.9	0.079
Albumin (mean ± SD) mg/L	3.7 ± 0.5	3.6 ± 0.4	0.068
Fracture classification (*n*, %)			
31A1	50 (31.5%)	10 (16.4%)	0.070
31A2	83 (51.9%)	40 (65.6%)	
31A3	27 (16.9%)	11 (18.0%)	
Lateral wall thickness (mean ± SD) mm	21.7 ± 6.6	20.4 ± 5.9	0.207
Neck shaft angle (mean ± SD) °	135.3 ± 7.8	133.9 ± 9.8	0.256
Medial cortical support (mean ± SD) mm	(+) 0.7 ± 3.4	(+) 0.8 ± 4.2	0.906
Anterior cortical support (mean ± SD) mm	(−) 1.1 ± 3.7	(−) 1.3 ± 4.8	0.679
CalTAD ^5^ (mean ± SD) mm	26.8 ± 5.8	26.6 ± 6.1	0.770
Parker’s ratio (AP) (mean ± SD) %	47.5 ± 7.8	48.8 ± 8.5	0.293
Fixation implant			
Extramedullary device	36 (22.5%)	10 (16.4%)	0.359
Intramedullary device	124 (77.5%)	51 (83.6%)	
Time to surgery (median, IQR) (d)	4 (2.5, 7)	5 (3, 8)	0.074

^1^ NMS, New Mobility Score; ^2^ Hb, hemoglobin; ^3^ CCI, Charlson comorbidity index; ^4^ BMI, body mass index; ^5^ CalTAD, calcar-referenced tip-apex distance.

**Table 2:** Multivariable fractional polynomial logistic regression model for predicting 1-year postoperative ambulatory status using patient’s baseline characteristics.

Predictors	Covariate Transformation			ß	95% CI	*p*-Value
	Terms	df	Formula			
Intercept				1.33	0.90, 1.76	
Male gender	Linear	1		0.21	−0.59, 1.00	0.608
Pre-fracture NMS ^1^	FP2	2	prenms^−2^—0.134	−85.70	−125.40, −46.00	<0.001
CCI ^2^	Linear	1	CCI—4.489	−0.42	−0.69, −0.16	0.002
BMI ^3^	Linear	1	BMI—21.806	−0.10	−0.20, −0.01	0.028

^1^ NMS, New Mobility Score; ^2^ CCI, Charlson comorbidity index; ^3^ BMI, body mass index.

**Table 3:** Multivariable logistic regression model after applying a multivariable fractional polynomial algorithm for predicting 1-year postoperative ambulatory status using prediction from the previous model and surgical related factors.

Predictors	Covariate Transformation			ß	95% CI	*p*-Value
	Terms	df	Formula			
Intercept				1.57	0.53, 2.61	0.003
Model 1 probability	Linear	1	model 1 prob—0.724	5.68	3.83, 7.53	<0.001
AO ^1^ 31A2	Linear	1		−0.74	−1.82, 0.34	0.182
AO 31A3	Linear	1		0.08	−1.36, 1.52	0.910
Lateral wall thickness	Linear	1	lateral wall—21.307	0.03	−0.04, 0.10	0.373
NSA ^2^	Linear	1	NSA—134.896	0.03	−0.02, 0.08	0.226
M-L ^3^ displacement	Linear	1	ML displacement—0.708	−0.04	−0.15, 0.06	0.377
A-P ^4^ displacement	Linear	1	AP displacement + 1.156	0.03	−0.06, 0.12	0.580
CalTAD ^5^	Linear	1	CalTAD—26.738	0.03	−0.04, 0.11	0.389
Parker’s ratio (AP)	Linear	1	Parker’s ratio—47.825	−0.01	−0.06, 0.05	0.790
Intramedullary device	Linear	1		−0.01	−1.26, 1.25	0.990

^1^ AO, AO/OTA Classification; ^2^ NSA, neck-shaft angle; ^3^ M-L, medio-lateral; ^4^ A-P, antero-posterior; ^5^ CalTAD, calcar-referenced tip-apex distance.

## Data Availability

The datasets used and analyzed during the current study are available from the corresponding author on reasonable request. The data are not publicly available due to their containing information that could compromise the privacy of research participants.

## Appendix B

**Table A1:** Predicted odds and likelihood ratio (LHR) in discriminating 1-year postoperative ambulatory status at each decile from preoperative model’s predictive odds.

Decile	Total	NMS ≥ 5	NMS < 5	Median Predicted Odds	Range of Predictive Odds		Log Odds	LHR	95% Confidence Interval		Log LHR
					Min	Max					
D1	22	3	19	0.21	<0.01	0.55	0.10	0.14	0.09	0.19	−7.04
D2	22	10	12	0.94	0.60	1.28	0.52	0.58	0.53	0.62	−0.57
D3	22	17	5	1.82	1.30	2.44	1.13	1.13	1.05	1.22	0.11
D4	22	18	4	3.20	2.54	3.57	1.88	1.91	1.82	2.00	0.64
D5	22	13	6	3.90	3.69	4.25	2.48	2.40	2.36	2.44	0.87
D6	22	15	7	4.64	4.28	5.04	3.23	2.85	2.80	2.90	1.05
D7	22	21	1	5.57	5.10	6.41	3.88	3.45	3.35	3.55	1.24
D8	22	20	2	7.25	6.49	7.91	4.88	4.41	4.30	4.52	1.48
D9	21	16	5	9.01	8.21	10.56	6.54	5.54	5.36	5.72	1.71
D10	24	24	0	13.08	10.68	25.90	11.15	8.97	7.87	10.07	2.16
Total	221	160	61								

Abbreviations: NMS, New Mobility Score; Min, Minimum; Max, Maximum; CI, Confidential interval; LHR+, Positive likelihood ratio; D, Decile.

**Table A2:** Predicted odds and likelihood ratio (LHR) in discriminating 1-year postoperative ambulatory status at each decile from intraoperative model’s predictive odds.

Decile	Total	NMS ≥ 5	NMS < 5	Median Predicted Odds	Range of Predicted Odds		Log Odds	LHR	95% Confidence Interval		Log LHR
					Min	Max					
D1	22	3	19	0.11	0.03	0.41	−2.14	0.10	0.07	0.13	−2.64
D2	22	10	12	0.87	0.49	1.21	−0.20	0.52	0.46	0.57	−0.69
D3	22	18	4	1.86	1.24	2.41	0.59	1.13	1.02	1.23	0.09
D4	22	12	10	3.02	2.50	3.52	1.12	1.88	1.81	1.95	0.63
D5	22	18	4	4.03	3.53	4.82	1.40	2.48	2.37	2.59	0.90
D6	22	14	8	5.28	4.88	5.73	1.67	3.23	3.15	3.30	1.17
D7	22	21	2	6.35	5.84	7.02	1.85	3.88	3.79	3.97	1.36
D8	22	21	1	7.97	7.07	8.77	2.08	4.88	4.76	5.00	1.58
D9	22	21	1	10.62	8.93	13.39	2.37	6.54	6.23	6.85	1.87
D10	23	23	0	18.05	13.47	30.46	2.88	11.15	10.11	12.19	2.39
Total	221	160	61								

Abbreviations: NMS, New Mobility Score; Min, minimum; Max, maximum; CI Confidential interval; LHR, Likelihood ratio; D, Decile.

## Appendix C

**Table A3:** A clinical application demonstration of the IT-AP tool.

Variable	Preoperative Model	Preliminary Adjustment	Final Adjustment
Pre-injury NMS ^1^	9		
CCI ^2^	4		
BMI ^3^ (kg/m^2^)	22.49		
Sex	male		
Configuration			
AO/OTA classification		31A1	31A1
Lateral wall thickness (mm)		33.9	33.9
NSA ^4^ (°)		124	129
Medial cortical support (mm)		5.4	0
Anterior cortical support (mm)		−6.3	0
CalTAD ^5^ (mm)		19.9	29.9
Parkes’s ratio (%)		63	42
Implant		Intramedullary device	Intramedullary device
Likelihood ratio	4.70	3.70	10.59
Prediction	More likely to ambulate	More likely to ambulate	Very likely to ambulate
Recommendation		Inadequate operation	Adequate operation

^1^ NMS, New Mobility Score; ^2^ CCI, Charlson comorbidity Index; ^3^ BMI, body mass index; ^4^ NSA, neck-shaft angle; ^5^ CalTAD, calcar-referenced tip-apex distance.
